# Referrals of young people by primary care: What proportion have neuro-developmental needs?

**DOI:** 10.1192/j.eurpsy.2026.11173

**Published:** 2026-07-31

**Authors:** S. Jones, L. Wood, Z. Mutty, M. Miller, J. Beezhold

**Affiliations:** 1 Mental Health Advice Support and Access; 2 Cambridgeshire Community Services NHS Trust; 3 Norwich Medical School, University of East Anglia, Norwich, United Kingdom

## Abstract

**Introduction:**

A new Mental Health Advice Support and Access Service (MHASA) was introduced to triage all referrals for people aged 0-25 years referred to mental health services. Following this triage process, patients are passed to the most appropriate service available. This study reports data regarding people referred who also have suspected neurodevelopmental disorders (NDD).

**Objectives:**

To report data regarding those referred with suspected NDD including referral numbers, gender, age, primary presenting needs, diagnostic status and outcome of triage.

**Methods:**

A random sample comprising 358 out of the 765 cases referred in November 2024 was analysed, based on statistical advice regarding sample size required to generate robust findings.

Referrals were analysed by gender, age, primary presenting needs, diagnostic status and outcome of triage.

**Results:**

Half of all the referrals had identified NDD concerns at the point of referral.

2/3 male referrals in the NDD identified group, as compared to 1/3 females.

61% of those identified with NDD are diagnosed or on a waiting list.

Peak age for referrals with NDD identified cases - at 12yrs and 15yrs. Educational transitions - 12yrs move to high school - - 15yrs at exam times.

Primary Presenting Needs identified at point of triage – half of the NDD identified referrals presented with Anxiety.

Only 27% of the NDD identified cases were referred on to a Specialist Mental Health service i.e. NSFT CAMHS/ Youth.

**Image 1:**
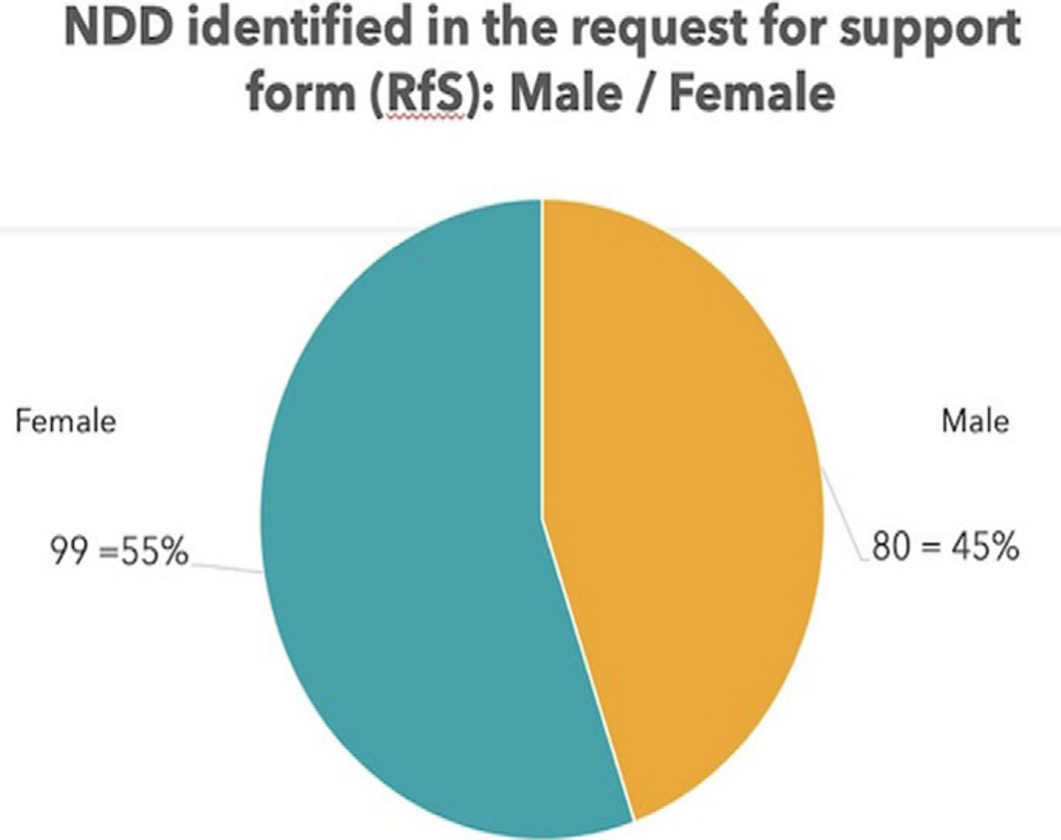

**Image 2:**
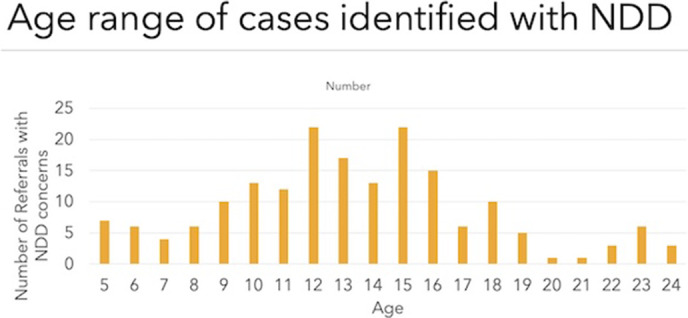
Image 2: Long description.

**Image 3:**
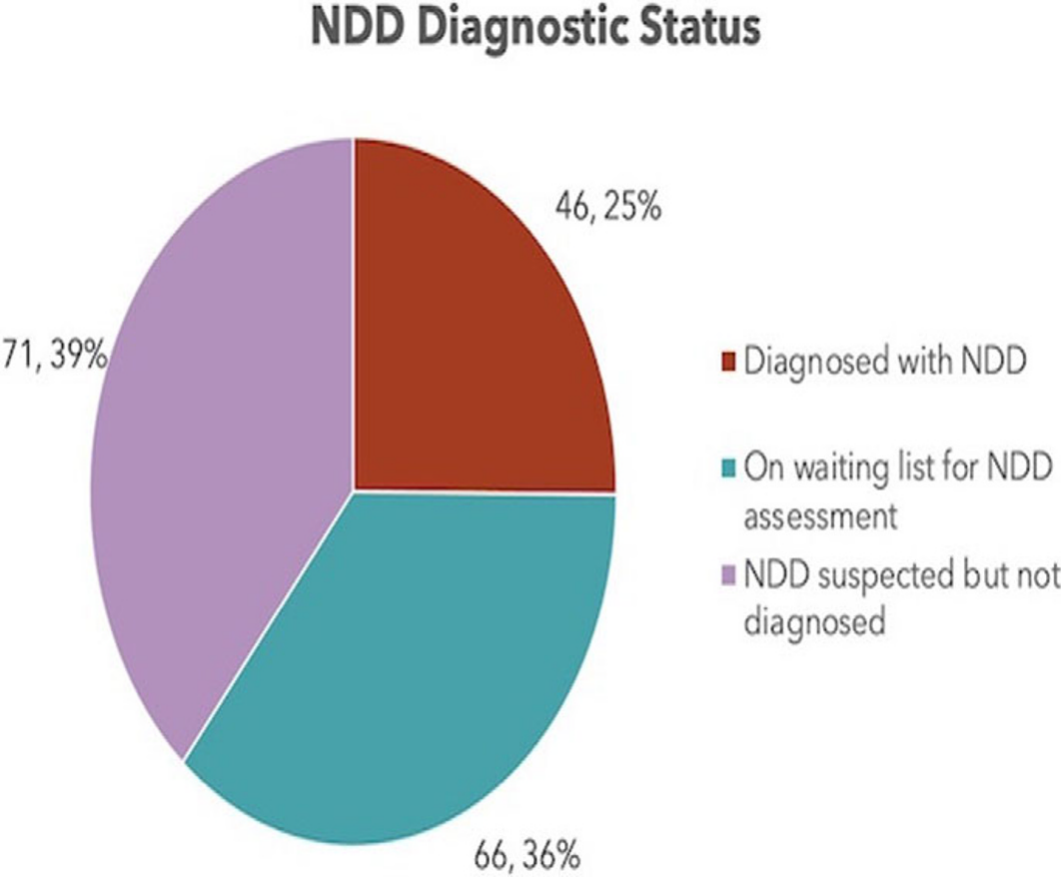

**Conclusions:**

These findings are significant new data regarding the prevalence and presentation of young people with suspected NDD who are referred to mental health services in England.

The findings strongly suggest that the numbers of young people referred with suspected NDD is higher than expected, with important implications for health care providers.

This will be relevant for health service commissioners and providers when planning to meet this need.

**Disclosure of Interest:**

None Declared

## Long descriptions

### Image 2: Long description

The vertical Y axis is labeled Number of Referrals with N D D concerns and ranges from 0 to 25 in increments of 5. The horizontal X axis is labeled Age and ranges from 5 to 24 in single-year increments. The data shows a bimodal distribution with two primary peaks.

* From age 5 to 7, referrals decrease from 7 to 4.

* From age 8 to 12, there is a steady increase, reaching the first peak of 22 referrals at age 12.

* Referrals dip to 17 at age 13 and 13 at age 14.

* A second peak of 22 referrals occurs at age 15.

* From age 16 to 21, there is a general downward trend, reaching a low of 1 referral at ages 20 and 21.

* A small final increase occurs at age 23 with 6 referrals, before dropping to 3 at age 24.

Navigate back to Image 2:.

